# Relationship between obesity and development of erosive reflux disease: A mediation analysis of the role of cardiometabolic risk factors

**DOI:** 10.1038/s41598-017-06845-1

**Published:** 2017-07-25

**Authors:** Hyuk Lee, Yaeji Lim, Sangah Chi, Yang Won Min, Byung-Hoon Min, Jun Haeng Lee, Poong-Lyul Rhee, Jae J. Kim

**Affiliations:** 1 Department of Medicine, Samsung Medical Center, Sungkyunkwan University School of Medicine, Seoul, Republic of Korea; 2 Department of Statistics, Pukyong National University, Pusan, Republic of Korea; 3 Biostatistics and Clinical Epidemiology Center, Samsung Medical Center, Sungkyunkwan University School of Medicine, Seoul, Republic of Korea

## Abstract

This study aimed to evaluate whether the relationship between obesity and erosive reflux disease (ERD) is mediated by cardiometabolic risk factors. This cohort study included subjects who underwent repeated endoscopy. To assess whether the association between body mass index (BMI) and ERD development was mediated by cardiometabolic parameters, multivariate Cox proportional hazard models were fitted for mediation analysis. Of 15,154 subjects with negative index endoscopy findings during health check-up, 11,686 subjects who underwent repeated endoscopy were included. During follow-up, 1,367 incident ERD events (11.7%) were observed. Without mediation effect, the multivariate adjusted hazard ratio of BMI was 1.21 (95% CI, 1.03–1.42) in overweight and 1.39 (95% CI, 1.19–1.62) in obese individuals compared to normal weight individuals. When the metabolic syndrome was included as a mediator, the hazard ratio became 1.19 (95% CI, 1.00–1.40) in overweight and 1.29 (95% CI, 1.10–1.52) in obese individuals. Both systolic blood pressure and triglyceride level were found to fully mediate the effect of BMI on ERD. Fasting glucose level was a partial mediator. The estimated percentage of total effect mediated by cardiometabolic risk factors was 35.4%. Cardiometabolic parameters partially or fully mediate the association between overweight and obesity and incident ERD.

## Introduction

Gastroesophageal reflux disease (GERD) is a common gastrointestinal disorder that frequently occurs in the primary care setting, with a high direct and indirect economic burden on the society^1, 2^. GERD is a multifactorial disease in which anatomical and functional factors play a role in pathogenesis^3^. Among various risk factors for erosive reflux disease (ERD), obesity has been considered an independent risk factor and it seems that the risk of developing ERD increases with increasing body mass index (BMI)^4–10^. Some meta-analyses revealed a positive association between BMI and the presence of ERD^11–13^. Although the exact pathophysiological mechanisms underlying this association have not been fully identified, it has been suggested that intra-abdominal pressure from visceral adiposity or esophageal peristaltic abnormalities might cause ERD in obese subjects^14–18^. Metabolic syndrome is characterized by visceral fat accumulation, dyslipidemia, hypertension, and hyperglycemia^19^, and all these factors have been suggested to correlate with the occurrence of ERD^20–23^. A meta-analysis showed that central adiposity is definitely associated with ERD, independent of BMI^24^. However, the mechanisms that link for example biochemical cardiometabolic parameters or blood pressure and ERD cannot be solely explained by the mechanical effect of obesity. In addition, whether coexisting metabolic syndrome is a necessary condition for the development of ERD in overweight and obese individuals remains controversial^25^. Indeed, metabolic syndrome parameters were reported to promote esophageal injury by creating pro-inflammatory and insulin-resistant milieu^26^.

In the present study, we tested whether metabolic syndrome, irrespective of obesity, was associated with increased risk of ERD. In addition, we aimed to evaluate whether the association between the obesity and ERD is mediated by cardiometabolic risk factors.

## Results

### Clinical characteristics of population

Table 1 shows baseline characteristics of the 11,686 individuals included in the study. This population was classified by BMI category and by the absence or presence of metabolic syndrome. Normal weight, overweight, and obesity were observed in 31.4%, 28.4%, and 40.1% of cases, respectively. Proportion of diagnosis of metabolic syndrome was 3.0%, 10.5%, and 27.7% in normal weight, overweight, and obese individuals, respectively. Characteristics of subjects classified by incident ERD are shown in Table 2. During a median (interquartile range) follow-up time of 3.6 (range, 2.4–5.7) years, ERD was diagnosed in 1,367 individuals (11.7%): 1215 (88.9%) individuals had LA-A grade, 140 (10.2%) had LA-B grade, and 12 (0.9%) individuals had LA-C grade. No difference was found in mean duration from index negative endoscopy to the onset of ERD among these grade subgroups (60.8 months, 62.7 months, and 55.7 months, *P* = 0.140). Significant differences were observed in baseline metabolic parameters, including BMI, waist circumference, and levels of serum high-density lipoproteins, triglycerides, and glucose among these groups. More importantly, a significant difference was found in change of almost all parameters from their respective baseline levels between ERD and control groups. The number of individual metabolic syndrome components in BMI subgroups is presented in Supplementary Table S1. Supplementary Tables S2–S4 demonstrate clinical characteristics according to sex. Incident ERD was diagnosed in 16.0% of male and 5.1% in female subjects. Among male participants, the frequency of metabolic syndrome was 2.8%, 7.8%, and 26.6% in subgroups with normal weight, overweight, and obesity, respectively. These frequencies among the female subjects were 3.1%, 15.2%, and 31.9%, respectively. Supplementary Table S5 shows the risk of ERD associated with individual components of metabolic syndrome. Fasting blood glucose level was the highest risk factor for ERD with a hazard ratio (HR) of 3.95 (95% CI, 3.01–5.18). Risk of ERD according to the number of components of metabolic syndrome is presented in Supplementary Table S6.

**Table 1:** Baseline characteristics of subjects according to body mass index categories and presence or absence of metabolic syndrome.

	Normal weight		Overweight		Obese	
	No MS	MS	No MS	MS	No MS	MS
Patients, No.	3565	110	2974	350	3387	1300
Age, median (IQR)	48 (43–54)	56 (49–62)	51 (46–57)	56 (50–62)	50 (46–56)	52 (47–59)
Male, %	35.1	32.7	65.4	47.1	79.6	75.2
BMI, median (IQR)	21 (20–22)	22 (21–22)	24 (23–24)	24 (23–24)	26 (25–27)	27 (26–29)
Waist circumference, median (IQR), cm	74 (70–79)	80 (76–83)	83 (79–86)	84 (81–87)	89 (86–93)	94 (88–98)
BP, median (IQR), mm Hg						
Systolic BP	108 (100–118)	125 (114–136)	113 (104–124)	125 (112–135)	117 (107–126)	126 (113–135)
Diastolic BP	67 (60–74)	76 (66–83)	70 (63–78)	75 (68–82)	72 (65–80)	78 (70–85)
Medication use, %						
Antihypertensive	9.3	44.6	17.5	44.9	21.6	52.3
Antiglycemic	2.9	27.3	3.8	18.3	3.7	16.2
Aspirin	6.1	20.9	11.7	20.9	12.6	21.8
Plasma levels, median (IQR)						
Total cholesterol, mg/dL	187 (166–208)	193 (166–215)	192 (172–215)	195 (168–219	193 (171–214)	196 (172–221)
Low-density lipoprotein	115 (98–135)	120 (100–145)	124 (107–145)	124 (103–146	127 (108–146)	126 (105–147)
High-density lipoprotein	62 (53–73)	45 (39–53)	56 (48–65)	45 (38–52)	52 (46–61)	44 (38–52)
Triglycerides, mg/dL	84 (66–114)	181 (147–220)	105 (77–141)	184 (153–240)	117 (88–150)	186 (150–252)
Glucose, mg/dL	86 (81–93)	104 (93–118)	89 (83–95)	103 (94–114)	90 (84–97)	103 (93–116)
HbA1c	5.2 (5.0–5.5)	5.8 (5.4–6.8)	5.3 (5.1–5.6)	5.7 (5.4–6.2)	5.4 (5.1–5.7)	5.7 (5.4–6.3)
Diabetes mellitus, %	4.2	37.3	5.6	24.3	5.1	20.8
Hypertension, %	9.6	47.3	18.5	48.0	22.5	55.6
Dyslipidemia, %	10.7	33.6	17.3	30.0	19.2	31.8
Smoking behavior, %						
None	71.6	66	49.6	58.9	38.7	38.2
Former	16.1	20.8	32.7	24.6	39.9	37.6
Current	12.4	13.2	17.8	16.4	21.4	24.3
Physical exercise, %	81.4	86.5	89.3	90.3	88.6	84.8

**Table 2:** Baseline characteristics of participants by incident erosive reflux disease.

	Erosive reflux disease		
	With event	Without event	*P* value
Patients, No.	1367	10319	
Age, median (IQR), y	51 (46–57)	50 (45–56)	0.621
Men, %	82.6	57.5	<0.001
BMI, median (IQR)	24 (23–26)	24 (22–26)	0.003
Change of BMI	0.25 ± 0.77	−0.13 ± 0.60	<0.001
Waist circumference, median (IQR), cm	87 (81–92)	83 (76–89)	<0.001
Change of waist circumference	0.60 ± 1.16	−0.10 ± 0.74	<0.001
BP, median (IQR), mm Hg			
Systolic BP	115 (105–126)	114 (104–126)	0.574
Diastolic BP	72 (65–80)	70 (63–78)	0.120
Change of systolic BP	5.40 ± 7.39	2.65 ± 7.08	0.043
Change of diastolic BP	3.40 ± 4.94	2.24 ± 4.80	<0.001
Medication use, %			
Antihypertensive	26.6	20.4	<0.001
Antiglycemic	7.3	5.3	0.007
Aspirin	14.3	11.4	0.006
Plasma levels, median (IQR)			
Total cholesterol, mg/dL	190 (170–211)	191 (169–214)	0.300
Change of total cholesterol	7.30 ± 10.30	−2.24 ± 10.25	<0.001
Low-density lipoprotein	122 (104–141)	122 (104–143)	0.437
Change of low-density lipoprotein	6.35 ± 7.37	−0.93 ± 7.44	<0.001
High-density lipoprotein	53 (45–61)	55 (47–65)	<0.001
Change of high-density lipoprotein	−0.05 ± 6.99	0.10 ± 6.52	<0.001
Triglycerides, mg/dL	126 (90–175)	107 (77–151)	<0.001
Change of triglycerides	11.60 ± 15.26	−4.09 ± 11.10	<0.001
Glucose, mg/dL	91 (84–100)	89 (83–97)	<0.001
Change of glucose	5.16 ± 6.11	−1.76 ± 6.67	<0.001
HbA1c	5.4 (5.1–5.7)	5.4 (5.1–5.6)	<0.001
Diabetes mellitus, %	11.3	7.1	<0.001
Hypertension, %	27.7	21.5	<0.001
Dyslipidemia, %	20.9	17.6	0.003
Smoking behavior			
None	33.1	54.8	<0.001
Former	37.6	28.9	
Current	29.3	16.3	
Physical exercise, %	88.8	85.8	0.011

### Risk of ERD according to body mass index category and the metabolic syndrome

**Figure 1:**
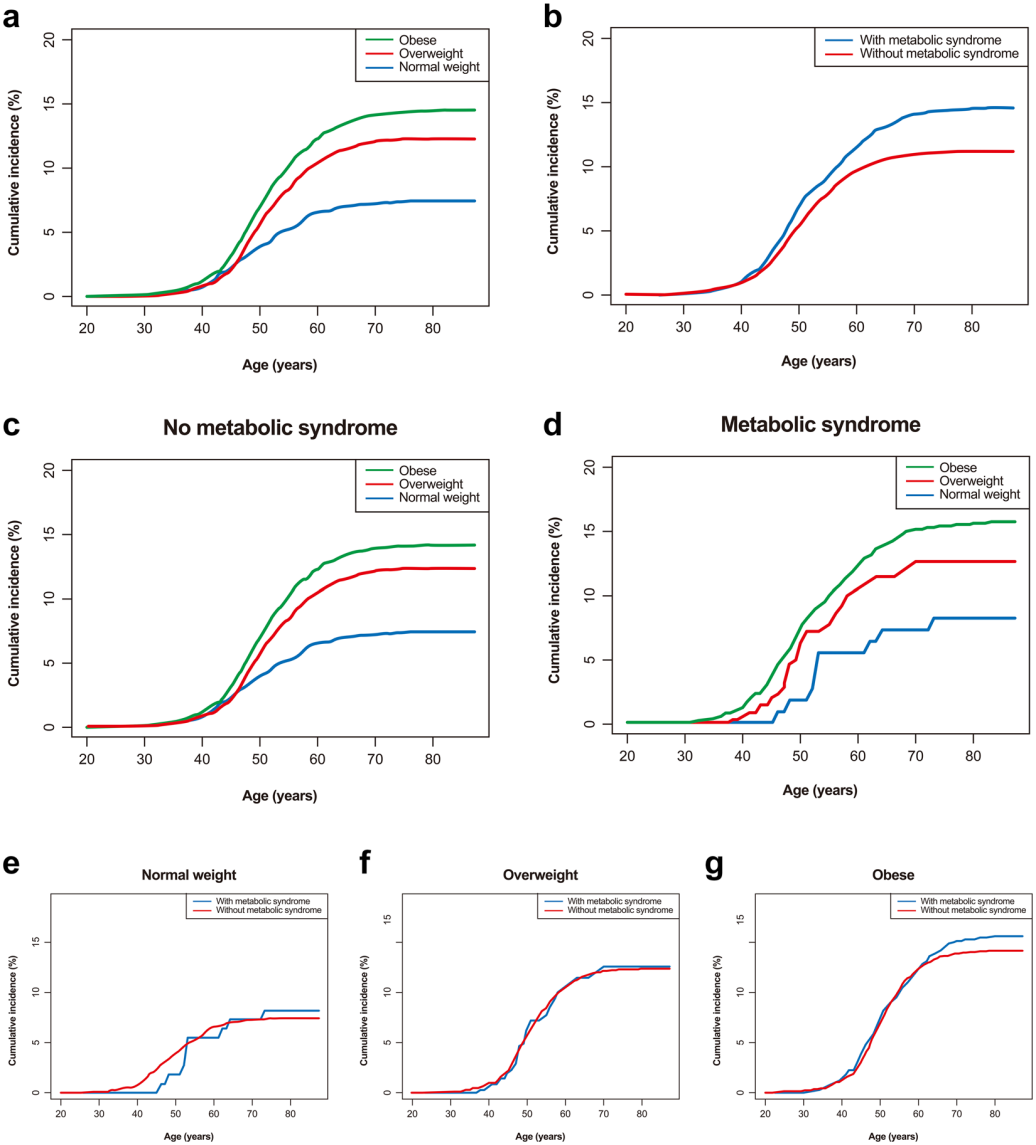
Risk of erosive reflux disease according to body mass index category (**a**) and absence/presence of the metabolic syndrome (**b**). Cumulative incidences of erosive reflux disease according to body mass index category in groups classified by absence (**c**)/presence of the metabolic syndrome (**d**). Cumulative incidences of erosive reflux disease according to absence/presence of the metabolic syndrome in groups classified by body mass index category (normal weight: (**e**), overweight: (**f**), obese: (**g**)).

The cumulative incidences of ERD were significantly higher in patients with overweight and obesity compared to individuals with normal weight (both log-rank *P* < 0.001) (Fig. 1a). Without mediation effect, multivariable adjusted HRs for ERD were 1.21 (95% CI, 1.03–1.42) in overweight and 1.39 (95% CI, 1.19–1.62) in subjects with obesity. In addition, the presence of metabolic syndrome was associated with increased cumulative incidences of ERD (log-rank *P* < 0.001) (Fig. 1b). Multivariable adjusted HR for ERD in subjects with metabolic syndrome was 1.44 (95% CI, 1.24–1.66).

**Figure 2:**
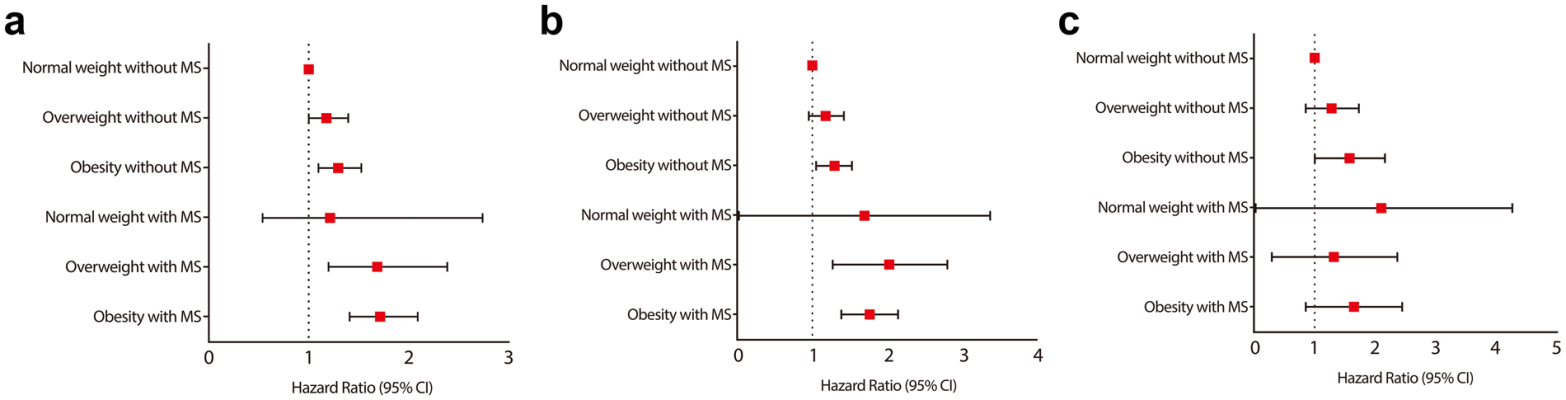
Risk of erosive reflux disease according to combination of body mass index category and the metabolic syndrome (**a**). Risk of erosive reflux disease according to combination of body mass index category and the metabolic syndrome in male (**b**) and female (**c**).

When individuals were divided into six groups according to their BMI category and metabolic syndrome, risk of ERD increased with higher BMI category in the absence of metabolic syndrome (Fig. 2a). For ERD, multivariable adjusted HRs were 1.19 (95% CI, 1.00–1.40) in overweight and 1.29 (95% CI, 1.10–1.52) in obese individuals without metabolic syndrome compared to normal weight subjects without metabolic syndrome. In addition, multivariable adjusted HRs were 1.22 (95% CI, 0.54–2.74) in normal weight, 1.69 (1.20–2.39) in overweight, and 1.71 (95% CI, 1.41–2.09) in obese subjects with metabolic syndrome. The difference of ERD incidence by presence of metabolic syndrome was more significant in males, whereas metabolic syndrome did not influence ERD incidence in females (Fig. 2b,c). In the subgroup of normal weight, contrary to overweight or obese individuals, metabolic syndrome was not associated with the incidence of ERD.

In the subgroup without metabolic syndrome, higher cumulative incidences of ERD were associated with higher BMI (log-rank *P* < 0.001) (Fig. 1c). However, in the subgroup with metabolic syndrome, an ambiguous increment of cumulative incidences of ERD according to BMI category was found (log-rank *P* = 0.293) (Fig. 1d). When classified for BMI category, the presence of metabolic syndrome within overweight and obese subjects was associated with increased cumulative incidences of ERD (log-rank *P* = 0.05 and *P* < 0.001, respectively) (Fig. 1e–g). Normal weight subgroup did not demonstrate a significant association between metabolic syndrome and ERD development (*P* = 0.20).

### Mediation analysis of the role of cardiometabolic risk factors

In mediation analysis, the percentage of excess risk mediated by metabolic syndrome in the association between BMI category and ERD incidence was 9.2%; that is, 9.2% of the associated effect size of BMI on risk of ERD is explained by metabolic syndrome (Table 3). Metabolic syndrome was a partial mediator in the relationship of obesity and ERD, which means that the HR of ERD reduced in absolute size, but was still different from zero when the mediator was introduced. Table 4 showed the excess risk of overweight and obesity mediated through different combinations of cardiometabolic risk factors. Among the individual metabolic risk parameters, SBP was the most important mediator for the association of overweight and obesity with ERD. The effect of BMI on ERD was fully mediated by TG and SBP. That is, the path from BMI to ERD became insignificant when these mediation variables were introduced. Using the bootstrapping-based mediation analysis it was estimated that 16.2% (*P* = 0.03) of the total effect of BMI on ERD was mediated by TG, and 13.3% (*P* = 0.01) of the total effect of BMI on ERD was mediated by SBP. The effect of BMI on ERD was partially mediated by FG. The bootstrapping-based mediation analysis estimated that the percentage of total effect mediated by FG was 9.5% (*P* = 0.01). The combination of TG and SBP, which are fully mediators, accounted for a high percentage of excess risk of BMI (31.9%). The collective effect mediated by these three mediators (TG, SBP, and FG) was 35.4% (*P* < 0.001).

**Table 3:** Percentage of excess risk mediated by body mass index or the metabolic syndrome.

Exposure	Mediator	Confounder adjusted		Confounder and mediator adjusted		p-value of mediation effect	Percentage of effect mediated (%)
		HR (95% CI)	p	HR (95% CI)	p		
Overweight	Metabolic syndrome	1.208 (1.025–1.422)	0.024	1.190 (1.010–1.402)	0.038	0.02	9.18 (4.19–25.62)
Obesity		1.387 (1.189–1.616)	<0.001	1.294 (1.103–1.517)	0.002

**Table 4:** HRs and excess risk of overweight and obesity mediated through different combinations of metabolic risk factors.

Mediator used	HR (95% CI)		p-value of mediation effect	Percentage of effect mediated (%)
	Overweight	Obese		
SBP	1.175 (0.997–1.384)	1.321 (1.130–1.543)	0.01	13.34
TG (log transformed)	1.162 (0.985–1.371)	1.300 (1.109–1.523)	0.03	16.19
HDL	1.185 (1.004–1.398)	1.349 (1.153–1.579)	0.12	8.65
FG (log transformed)	1.184 (1.005–1.394)	1.321 (1.132–1.541)	0.01	9.47
SBP and TG	1.136 (0.962–1.341)	1.249 (1.064–1.467)	<0.001	31.91
SBP, TG, and FG	1.130 (0.957–1.333)	1.219 (1.038–1.431)	<0.001	35.42

## Discussion

In our study that involved a large-scale population that underwent regular health check-ups in the form of repeated screening endoscopy, we estimated that nearly one-third of excess risk for endoscopic ERD due to high BMI was mediated through three cardiometabolic risk factors: SBP, TG level, and FG level. The most important mediator was TG, accounting 16% of the excess risk. Compared to the normal weight, overweight or obese status was associated with an increased risk of ERD, with obesity having a greater effect than overweight.

Nearly all epidemiologic studies have found an association between increasing BMI and symptoms of GERD^4, 8–10, 12, 13, 27, 28^. Although the precise pathophysiological link between obesity and GERD has not been fully elucidated, multiple mechanisms have been implicated to account for this observation. Individual studies have variably found reduced lower esophageal sphincter (LES) pressure, increased frequency of transient LES relaxations, increased prevalence of hiatal hernia, increased prevalence of esophageal motor disorders, elevated intragastric pressure, and disorders of gastric accommodations in obese subjects^29^. In addition, abdominal adipose tissue, especially visceral fat, is also considered metabolically active, secreting inflammatory mediators, cytokines, and insulin-like growth factors, leading to a systemic inflammatory and insulin resistant state^18, 24, 26^. Some strong evidences support the positive relationship between the metabolic syndrome and ERD^30, 31^. However, with current epidemiologic evidence, the degree of contribution of each mechanical and metabolic factor to the ERD development remains unclear.

In statistics, a mediation model explains the process between an independent variable, X, and an outcome variable via the inclusion of a third hypothetical variable, mediator variable, M. It is considered that it “mediates” the relationship between X and Y. Baron *et al*. and Imai *et al*.^32, 33^ proposed the following three steps in establishing mediation: (1) The relation between the predictor, X, and the outcome, Y (this step establishes that there is an effect that may be mediated); (2) The predictor, X, is related to the mediator, M (this step involves treating the mediator as if it were an outcome variable); (3) The mediator affects the outcome, Y (that is, Y is used as the outcome variable in the regression equation and X and M are used as predictors). If these three steps are met, then partial mediation is demonstrated. If the effect of X on Y controlling for M is zero, it is suggested that the variable M completely mediates the X-Y relationship. Since, we used survival outcome variable, ERD, we performed Cox proportional regression model for mediation analysis^34^. Imai *et al*. described in detail the R package, “mediation”^33^.

In the mediation analysis of our study, a small proportion of the increased risk observed for BMI was contributed by metabolic syndrome. When excluding the waist circumference representative of abdominal obesity, the effect of metabolic syndrome on development of ERD was indirect with a percentage of excess risk of 9.2%. Regarding the individual components of metabolic syndrome, hypertension suggested by SBP was the most important mediator of both overweight-ERD and obesity-ERD association. Our findings regarding cardiometabolic risk factors are supported by previous studies on metabolic syndrome components and ERD^35–37^. They reported a significant relationship between ERD and metabolic parameters, such as dyslipidemia (especially hypertriglyceridemia), hyperglycemia, and hypertension. The importance of triglycerides level for the risk of ERD has been reported by some studies^23, 38^. A study on the Barrett esophagus and metabolic syndrome revealed that metabolic syndrome was independently of obesity associated with Barrett esophagus by a reflux-independent pathway^39^. However, whether the pathogenesis of esophagitis development is local, mechanical, or systemic remains unclear.

To the best of our knowledge, this is the first application of mediation analysis to estimate the role as full or partial mediators of cardiometabolic parameters in the relationship of obesity and incidence of ERD. This analysis provided a quantitative assessment of the extent to which the effect of overweight and obesity on ERD was mediated by inflammatory parameters. Considering the level of contribution of individual metabolic biomarkers, the results of this study support the therapeutic targeting toward metabolic syndrome, such as antihypertensive, antilipidemic, or hypoglycemic management, in patients with ERD. Especially life style modification can be considered an important management option for metabolic syndrome, not just for obesity resolution, but also because of cardiometabolic parameter improvement.

We acknowledge some potential limitations of this analysis. First, interobserver variations were not evaluated in the endoscopic diagnosis of ERD. However, all investigators in this study were highly experienced in endoscopic diagnosis. Second, there seemed to be a recall error in self-reported risk factors, although this error is unlikely to be biased by outcomes, because all baseline data were collected before endoscopy. Finally, selection bias might exist, because the samples were created just from routine health check-up population.

In conclusion, individual cardiometabolic parameters, which are the components of metabolic syndrome, such as SBP, TG level, and FG level, partially or fully mediate the association between overweight and obesity and incident ERD. Prospective studies to confirm clinical relevance of correction of metabolic risk factor for improvement of ERD are warranted.

## Methods

### Design Overview

We performed a retrospective cohort analysis of database records for subjects who entered the health check-up program for upper GI cancer at the Center for Health Promotion, Samsung Medical Center in Korea. This comprehensive health-screening program included anthropometric measurements, annual or biennial endoscopy, various laboratory studies, and an epidemiological questionnaire on lifestyle factors, medication, and chronic diseases. Health check-up costs were voluntarily supported by subjects or were partly supported by an affiliated company. The study was approved by the institutional review board of Samsung Medical Center, and due to the retrospective nature of the study, the requirement for informed consent was waived.

### Study Sample

**Figure 3:**
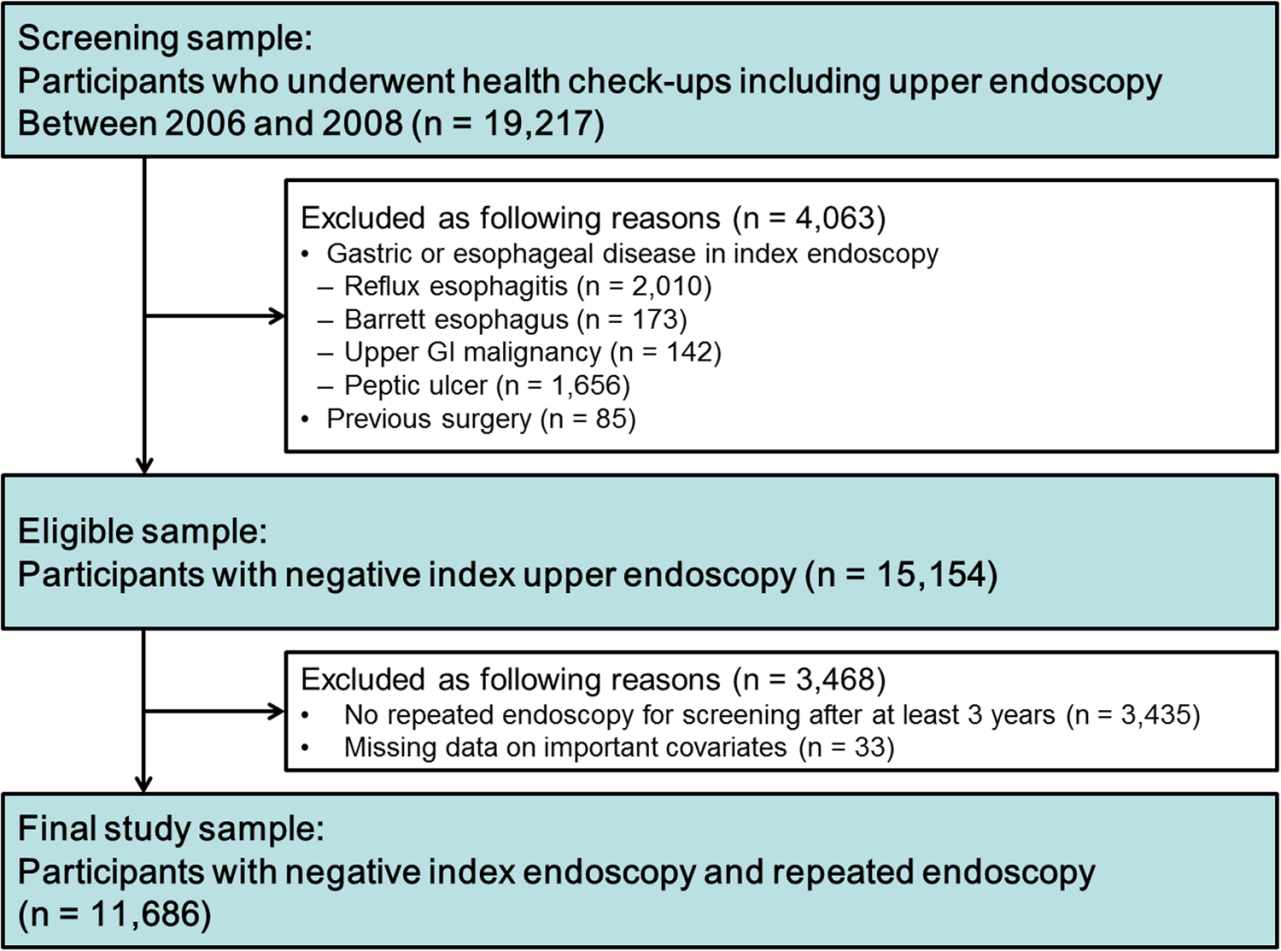
Flow diagram of study participants.

In total, 19,217 subjects who underwent first upper endoscopic screening examination between January 2006 and December 2008 were enrolled (Fig. 3). All subjects were asymptomatic at the time of baseline index endoscopy. Subjects were excluded if their index baseline endoscopy showed ERD, Barrett esophagus, malignant disease of the upper GI tract, and active or healing peptic ulcer disease. In addition, subjects with prior gastroesophageal surgery, partially completed epidemiological questionnaire, or missing records were also excluded. The subjects from this population with negative index endoscopy were included if they underwent repeated endoscopy after an interval of at least 3 years. This interval time from most of non-erosive state to erosive disease was determined based on previous data from a kinetic curve showing the development to erosive reflux disease (Supplementary Fig. S1)^40^. The final cohort included 11,686 subjects, and none of them needed additional medical treatment, as they did not have clinically relevant GI symptoms at the time of index endoscopy.

### Endpoint, definitions, and covariates

The endpoint was ERD development detected during the secondary endoscopy after a negative index endoscopy for screening. ERD was diagnosed if definite erosions (mucosal breaks) were present, and was classified according to the Los Angeles classification system^41^. All subjects had their BMI, body fat, and waist circumference measured by previously described techniques^42^. Weight and height were measured in the morning with subjects wearing light clothing, but no shoes, and BMI was calculated as weight in kilograms divided by the square of the height in meters. The guidelines presented by the World Health Organization of the Asia Pacific Region and the Korean Society for the Study of Obesity present the following definitions: normal (≤22.9 kg/m2), overweight (23–24.9 kg/m2), and obese (≥25 kg/m2)^43, 44^. The waist circumference was measured midway between the lower border of the rib cage and the iliac crest when subjects were standing at the end of normal expiration. We measured blood pressure and blood markers, such as levels of fasting glucose (FG), triglyceride (TG), low-density lipoprotein cholesterol (LDL), and high-density lipoprotein (HDL) cholesterol. Blood samples were collected from the antecubital vein after overnight fasting. Total cholesterol, LDL, HDL, TG, and FG levels were measured using enzymatic or colorimetric methods. Serum glucose levels were measured using the hexokinase/glucose-6-phosphate dehydrogenase method with a Hitachi 7600 Modular Dp-110 auto-analyzer (Hitachi, Tokyo, Japan). The average inter-assay and intra-assay coefficients of variation for quality control were 6.5% and 2.1% for FG, and 2.5% and 2.5% for glycated hemoglobin A1c (HbA1c) levels, respectively. Systolic blood pressure (SBP) and diastolic blood pressure were measured after a rest of 5 min in a sitting position. Structured questionnaires included self-reported comorbidities (diabetes, hypertension, or dyslipidemia). Subjects were classified as current smoker, former smoker, or never smoker. Regular exercise was defined as performing physical exercise of at least moderate intensity >3 times per week, for at least 30 min each time. Medication history of antihypertensive agents, aspirin, and non-steroidal anti-inflammatory drug use was also collected.

### Metabolic syndrome

We used a slightly modified version of the harmonized metabolic syndrome definition, because all components included are easily measured in clinical practice. Metabolic syndrome was defined when 3 or more of the following 5 metabolic abnormalities were present: (1) waist circumference at least 94 cm in men and at least 80 cm in women, (2) systolic blood pressure at least 130 mm Hg and/or diastolic blood pressure at least 85 mm Hg and/or antihypertensive treatment, (3) nonfasting plasma TG level at least 1.7 mmol/L, (4) HDL cholesterol level less than 1.04 mmol/L in men and less than 1.29 mmol/L in women, and (5) registry-documented diagnosis of diabetes mellitus and/or self-reported diabetes mellitus and/or antidiabetic treatment and/or nonfasting plasma glucose level more than 11.1 mmol/L^45^.

### Statistical analysis

The distribution of continuous variables was checked for normality before the analysis; fasting insulin and TG/HDL-cholesterol were normalized by a natural logarithm transformation. Partial correlation coefficients controlling for age were estimated to examine the relationship between BMI and ERD with cardiometabolic risk factors.

To examine whether the association between BMI and ERD was mediated by cardiometabolic parameters, Cox proportional hazard regression models were fitted based on the procedures outlined by Baron and Kenny^32^. The first equation regressed the dependent variable (ERD) on the independent variable (BMI). The second equation regressed the dependent variable (systolic blood pressure, log TG/HDL-c ratio, or fasting glucose) on the independent variable. The third equation regressed the dependent variable on both the independent and mediator variables.

The following criteria were used to establish mediation: 1) the independent variable must be significantly related to the mediator; 2) the independent variable must be significantly related to the dependent variable; 3) the mediator must be significantly related to the dependent variable; and 4) the association between the independent and dependent variable must be attenuated when the mediator is included in the regression model. In addition, we tested the significant of the mediation effect using bootstrapping, a resampling method that can be used to analyze the indirect effect^33, 46^.

A bilateral criterion for statistical significance of *P* ≤ 0.05 was used. Statistical analysis was performed using SAS software (version 9.4; SAS Institute Inc., Cary, NC, USA) and R statistical software (The R Project for Statistical Computing; http://www.cran.r-project.org/). Differences with a *P*-value < 0.05 were considered statistically significant.

## Electronic supplementary material

Supplementary Information
