# Supplementary material for: Relationship between obesity and development of erosive reflux disease: A mediation analysis of the role of cardiometabolic risk factors
Source: Sci Rep. 2017 Jul 25;7:6375. doi: 10.1038/s41598-017-06845-1 (PMC5527011; doi:10.1038/s41598-017-06845-1)

**Relationship between obesity and development of erosive reflux disease: A mediation analysis of the role of cardiometabolic risk factors**

Hyuk Lee, M.D, Ph.D.^1^**^*^**, Yaeji Lim, Ph.D.^2^**^*^**, Sangah Chi, B.S.^3^, Yang Won Min, M.D, Ph.D.^1^, Byung-Hoon Min, M.D, Ph.D.^1^, Jun Haeng Lee, M.D, Ph.D.^1^, Poong-Lyul Rhee, M.D, Ph.D.^1^, Jae J. Kim, M.D, Ph.D.^1^

^*^ These authors (Hyuk Lee and Yaeji Lim) contributed equally to this study.

^1^Department of Medicine, Samsung Medical Center, Sungkyunkwan University School of Medicine, Seoul, Republic of Korea, ^2^Department of Statistics, Pukyong National University, Pusan, Republic of Korea, ^3^Biostatistics and Clinical Epidemiology Center, Samsung Medical Center, Sungkyunkwan University School of Medicine, Seoul, Republic of Korea

Supplementary Table S1. Prevalent metabolic syndrome and number of metabolic syndrome components by body mass index category

|  | Normal | Overweight | Obesity |
| --- | --- | --- | --- |
| Number of subjects | 3675 | 3324 | 4687 |
| Metabolic syndrome, n(%) | 110 ( 3.0 ) | 350 ( 10.5 ) | 1300 ( 27.7 ) |
| Number of metabolic syndrome components, n(%) |  |  |  |
| 0 | 2191 ( 59.5 ) | 1128 ( 33.9 ) | 749 ( 16.0 ) |
| 1 | 1022 ( 27.8 ) | 1186 ( 35.7 ) | 1314 ( 28.0 ) |
| 2 | 352 ( 9.6 ) | 660 ( 19.9 ) | 1324 ( 28.2 ) |
| 3 | 89 ( 2.4 ) | 269 ( 8.1 ) | 882 ( 18.8 ) |
| 4 | 21 ( 0.6 ) | 68 ( 2.0 ) | 329 ( 7.0 ) |
| 5 | 0 ( 0 ) | 13 ( 0.4 ) | 89 ( 1.9 ) |

Supplementary Table S2. Baseline characteristics of male subjects by body mass index categories and presence/absence of the metabolic syndrome

|  | Normal weight | | Overweight | | Obese | |
| --- | --- | --- | --- | --- | --- | --- |
|  | no MS | MS | No MS | MS | No MS | MS |
| Patients, No. | 1250 | 36 | 1944 | 165 | 2696 | 977 |
| Age, median (IQR), y | 51 ( 45 - 58 ) | 55 ( 50 - 59 ) | 51 ( 46 - 57 ) | 55 ( 48 - 60 ) | 50 ( 46 - 56 ) | 51 ( 46 - 58 ) |
| BMI, median (IQR) | 21 ( 21 - 22 ) | 22 ( 21 - 22 ) | 24 ( 23 - 24 ) | 24 ( 23 - 24 ) | 26 ( 25 - 27 ) | 27 ( 26 - 29 ) |
| Waist circumference, median (IQR), cm | 79 ( 76 - 83 ) | 82 ( 79 - 85 ) | 85 ( 82 - 88 ) | 87 ( 84 - 90 ) | 90 ( 87 - 93 ) | 95 ( 92 - 99 ) |
| Blood pressure, median (IQR), mm Hg | | | | | | |
| Systolic | 111 (102-121) | 125 (113 - 135) | 115 ( 105 - 125 ) | 124 ( 113 - 135 ) | 117 ( 108 - 126 ) | 126 ( 113 - 135 ) |
| Diastolic | 70 ( 64 -77 ) | 76 ( 70 - 83 ) | 72 ( 65 - 80 ) | 77 ( 72 - 85 ) | 73 ( 66 - 80 ) | 79 ( 71 - 86 ) |
| Medication use, % |  |  |  |  |  |  |
| Antihypertensive | 12.7 | 41.7 | 19.3 | 46.7 | 22.3 | 52.5 |
| Antiglycemic | 5.7 | 36.1 | 5.2 | 23 | 4.2 | 15.8 |
| Aspirin | 10.1 | 22.2 | 14.4 | 24.2 | 14 | 23.5 |
| Plasma levels, median (IQR) | | | | | | |
| Total cholesterol, mg/dL | 183 (165-205) | 187 (156 - 213) | 189 ( 169 - 212 ) | 185 ( 162 - 208 ) | 192 ( 170 - 212 ) | 195 ( 171 - 220 ) |
| Low-density lipoprotein | 118 (100-136) | 113 ( 98 - 134 ) | 124 ( 106 - 143 ) | 117 ( 103 - 137 ) | 127 ( 108 - 146 ) | 125 ( 104 - 145 ) |
| High-density lipoprotein | 57 ( 49 - 66 ) | 39 ( 35 - 50 ) | 53 ( 47 - 61 ) | 42 ( 37 - 51 ) | 51 ( 45 - 59 ) | 43 ( 37 - 51 ) |
| Triglycerides, mg/dL | 97 (73 - 129) | 198 (167 - 264) | 113 ( 83 - 151 ) | 192 ( 159 - 249 ) | 122 ( 93 - 158 ) | 193 ( 155 - 264 ) |
| Glucose, mg/dL | 88 ( 82 - 96 ) | 106 (101 - 124) | 89 ( 84 - 97 ) | 106 ( 101 - 119 ) | 90 ( 85 - 97 ) | 104 ( 94 - 117 ) |
| HbA1c | 5.3 (5.1 - 5.6 ) | 5.9 (5.5 - 6.5) | 5.3 ( 5.1 - 5.6 ) | 5.7 ( 5.4 - 6.5 ) | 5.4 ( 5.1 - 5.7 ) | 5.7 ( 5.4 - 6.3 ) |
| Diabetes mellitus, % | 8.3 | 47.2 | 7.6 | 30.9 | 5.8 | 21.2 |
| Hypertension, % | 13 | 47.2 | 20.8 | 51.5 | 23.1 | 56.7 |
| Dyslipidemia, % | 14 | 47.2 | 19.2 | 32.7 | 20.8 | 33.6 |
| Smoking behavior |  |  |  |  |  |  |
| None | 30.9 | 13.9 | 25.7 | 17.3 | 25.2 | 20.3 |
| Former | 38.8 | 50 | 47.9 | 51.2 | 48.5 | 48.1 |
| Current | 30.3 | 36.1 | 26.4 | 31.5 | 26.4 | 31.6 |
| Physical exercise, % | 88.2 | 90.9 | 91.4 | 92.9 | 90 | 86.1 |

Supplementary Table S3. Baseline characteristics of female subjects by body mass index categories and presence/absence of the metabolic syndrome

|  | Normal weight | | Overweight | | Obese | |
| --- | --- | --- | --- | --- | --- | --- |
|  | no MS | MS | No MS | MS | No MS | MS |
| Patients, No. | 2315 | 74 | 1030 | 185 | 691 | 323 |
| Age, median (IQR), y | 47 ( 42 - 52 ) | 58 ( 50 - 63 ) | 50 ( 46 - 55 ) | 57 ( 51 - 62 ) | 52 ( 47 - 59 ) | 55 ( 48 - 61 ) |
| BMI, median (IQR) | 21 ( 20 - 22 ) | 22 ( 21 - 22 ) | 23 ( 23 - 24 ) | 24 ( 23 - 24 ) | 26 ( 25 - 27 ) | 26 ( 25 - 28 ) |
| Waist circumference, median (IQR), cm | 72 ( 68 - 75 ) | 78 ( 74 - 82 ) | 77 ( 74 - 80 ) | 82 ( 80 - 84 ) | 83 ( 80 - 87 ) | 86 ( 83 - 91 ) |
| Blood pressure, median (IQR), mm Hg | | | | | | |
| Systolic | 106 ( 98 - 117 ) | 126 ( 114 - 137 ) | 111 ( 102 - 122 ) | 125 ( 111 - 135 ) | 115 ( 106 - 127 ) | 125 ( 113 - 135 ) |
| Diastolic | 65 ( 60 - 72 ) | 74.5 ( 65 - 82 ) | 68 ( 60 - 75 ) | 72 ( 66 - 79 ) | 70 ( 62 - 76 ) | 74 ( 66 - 81 ) |
| Medication use, % |  |  |  |  |  |  |
| Antihypertensive | 7.5 | 46.0 | 14.0 | 43.2 | 19.1 | 51.7 |
| Antiglycemic | 1.4 | 23.0 | 1.3 | 14.1 | 1.7 | 17.6 |
| Aspirin | 3.9 | 20.3 | 6.8 | 17.8 | 7.1 | 16.4 |
| Plasma levels, median (IQR) | | | | | | |
| Total cholesterol, mg/dL | 188 ( 167 - 211 ) | 195 ( 170 - 218 ) | 197 ( 176 - 221 ) | 201 ( 176 - 226 ) | 198 ( 176 - 219 ) | 201 ( 179 - 225 ) |
| Low-density lipoprotein | 114 ( 97 - 134 ) | 121 ( 104 - 146 ) | 125 ( 108 - 147 ) | 132 ( 104 - 155 ) | 127 ( 108 - 146 ) | 132 ( 109 - 152 ) |
| High-density lipoprotein | 65 ( 56 - 75 ) | 47 ( 43 - 55 ) | 61 ( 53 - 70 ) | 46 ( 42 - 54 ) | 60 ( 53 - 68 ) | 48 ( 43 - 57 ) |
| Triglycerides, mg/dL | 79 ( 62 - 106 ) | 177 ( 132 - 197 ) | 93 ( 69 - 124 ) | 176 ( 126 - 231 ) | 97 ( 75 - 121 ) | 164 ( 122 - 209 ) |
| Glucose, mg/dL | 85 ( 80 - 91 ) | 104 ( 89 - 114 ) | 87 ( 82 - 93 ) | 101 ( 89 - 109 ) | 88 ( 83 - 94 ) | 101 ( 90 - 112 ) |
| HbA1c | 5.2 ( 5.0 - 5.5 ) | 5.7 ( 5.4 - 6.8 ) | 5.3 ( 5.1 - 5.6 ) | 5.7 ( 5.4 - 6.2 ) | 5.4 ( 5.2 - 5.7 ) | 5.8 ( 5.4 - 6.4 ) |
| Diabetes mellitus, % | 1.9 | 32.4 | 1.8 | 18.4 | 2.3 | 19.5 |
| Hypertension, % | 7.7 | 47.3 | 14.0 | 44.9 | 19.8 | 52.3 |
| Dyslipidemia, % | 8.9 | 27.0 | 13.5 | 27.6 | 13.2 | 26.3 |
| Smoking behavior |  |  |  |  |  |  |
| None | 93.9 | 92.9 | 95.6 | 96.7 | 93.4 | 94.8 |
| Former | 3.6 | 5.7 | 3.3 | 0.6 | 5.3 | 4.3 |
| Current | 2.6 | 1.4 | 1.1 | 2.8 | 1.4 | 1.0 |
| Physical exercise, % | 77.9 | 84.5 | 85.5 | 88.0 | 83.1 | 81.2 |

Supplementary Table S4. Baseline characteristics of participants by incident erosive reflux disease stratified by sex

|  | Male | | Female | |
| --- | --- | --- | --- | --- |
|  | Erosive reflux disease | | Erosive reflux disease | |
|  | With event | Without event | With event | Without event |
| Patients, No. | 1129 | 5933 | 238 | 4386 |
| Age, median (IQR), y | 51 ( 46- 57 ) | 51 ( 46- 57) | 51( 45- 57) | 49 ( 44- 55) |
| Men, % | 100 | 100 | 0 | 0 |
| BMI, median (IQR) | 25 ( 23- 26 ) | 25 ( 23- 26 ) | 23 ( 21-25 ) | 22 ( 21- 24 ) |
| Waist circumference, median (IQR), cm | 88 ( 84- 93 ) | 87 ( 83- 92 ) | 77 ( 72-83 ) | 75 ( 71- 81) |
| Blood pressure, median (IQR), mm Hg |  |  |  |  |
| Systolic | 115 (106-127) | 117 (107-127) | 112 (100-125) | 110 (100-122) |
| Diastolic | 73 (66-80) | 73 (66-80) | 68 ( 60-75 ) | 67 ( 60-75 ) |
| Medication use, % |  |  |  |  |
| Antihypertensive | 27.2 | 24.1 | 23.5 | 15.3 |
| Antiglycemic | 8.3 | 6.7 | 2.5 | 3.4 |
| Aspirin | 15.9 | 14.8 | 6.7 | 6.7 |
| Plasma levels, median (IQR) |  |  |  |  |
| Total cholesterol, mg/dL | 190 (169-210) | 190 (168-212) | 193 (172-218 ) | 192 (171-216) |
| Low-density lipoprotein | 122 (104-140) | 124 (105-144) | 121 (100-143) | 120 (102-142) |
| High-density lipoprotein | 51 (44-59) | 51 (44- 60) | 61 ( 54-73 ) | 61 ( 52-72 ) |
| Triglycerides, mg/dL | 133 (95-183) | 122 ( 88-170) | 98 ( 71-130) | 91 ( 68-125 ) |
| Glucose, mg/dL | 92 (85-102) | 91 ( 85-100 ) | 87 (82-93) | 87 ( 81-94 ) |
| HbA1c | 5.4 (5.1-5.8) | 5.4 (5.1- 5.7) | 5.3 ( 5.1-5.6 ) | 5.3 ( 5.1-5.6 ) |
| Diabetes mellitus, % | 12.8 | 9.1 | 4.6 | 4.3 |
| Hypertension, % | 28.5 | 25.7 | 24.0 | 15.7 |
| Dyslipidemia, % | 22.7 | 21.2 | 12.6 | 12.8 |
| Smoking behavior |  |  |  |  |
| None | 20.9 | 26.2 | 92.6 | 94.5 |
| Former | 44.8 | 47.0 | 2.6 | 3.8 |
| Current | 34.3 | 26.8 | 4.8 | 1.8 |
| Physical exercise, % | 89.6 | 89.6 | 85.0 | 80.9 |

Supplementary Table S5. Risk of erosive reflux disease by components of the metabolic syndrome and other risk factors individually

|  | Erosive reflux disease | |
| --- | --- | --- |
|  | HR (95% CI) | p-value |
| Components of the metabolic syndrome |  |  |
| Waist circumference (per 10 cm increase) | 1.376 (1.296-1.459) | <0.001 |
| Systolic blood pressure (per 10 mm Hg increase) | 1.108 (1.071-1.147) | <0.001 |
| Plasma triglycerides (per 1 mmol/l increase) | 1.592 (1.439-1.761) | <0.001 |
| High-density lipoprotein cholesterol (per 1 mmol/l increase) | 0.984 (0.980-0.988) | <0.001 |
| Fasting blood glucose (per 2 mmol/l increase) | 3.951 (3.012-5.182) | <0.001 |
| Other risk factors |  |  |
| Current smoking | 1.773 (1.574-1.998) | <0.001 |
| Physical exercise | 1.165 (0.975-1.392) | 0.092 |
| Total cholesterol, mg/dL | 1.002 (1.000-1.004) | 0.023 |
| Low-density lipoprotein | 0.999 (0.997-1.001) | 0.219 |

Hazard ratios with 95% confidence interval from a Cox regression model

Supplementary Table S6. Risk of erosive reflux disease according to the number of components of the metabolic syndrome

| Number of metabolic syndrome components | Subjects | Erosive reflux disease | HR(95%CI) |
| --- | --- | --- | --- |
| 0 | 4068 | 376 | 1 |
| 1 | 3522 | 437 | 1.2125 (1.0493-1.4009) |
| 2 | 2336 | 297 | 1.2474 (1.0616-1.4657) |
| 3 | 1240 | 184 | 1.4944 (1.2360-1.8068) |
| 4 | 418 | 59 | 2.2407 (1.6869-2.9764) |
| 5 | 102 | 13 | 1.8899 (1.0591-3.3725) |

**Supplementary Figure S1.** Plot of study design


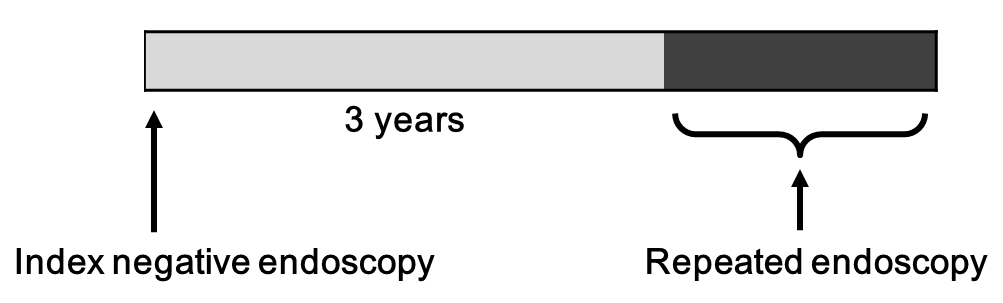

Supplement: Supplementary file 1 — Supplementary Information [file 41598_2017_6845_MOESM1_ESM.doc]
